# DNA barcoding and blood meal profiling of Ethiopian mosquitoes (Diptera: Culicidae): insights into species identification and host preferences

**DOI:** 10.1186/s13071-025-07135-w

**Published:** 2025-11-28

**Authors:** Samson Leta, Tesfaye Mulatu, Bekele Yalew, Tesfaye Rufael Chibssa, Jan Paeshuyse

**Affiliations:** 1 Laboratory of Host-Pathogen Interaction in Livestock, Division of Animal and Human Health Engineering, Department of Biosystems, KU Leuven, 3001 Leuven, Belgium; 2 Department of Biomedical Sciences, College of Veterinary Medicine and Agriculture, Addis Ababa University, P.O. Box 34, Bishoftu, Ethiopia; 3 Animal Health Institute (AHI), P.O. Box 04, Sebeta, Ethiopia

**Keywords:** Arbovirus, *COI*, DNA barcoding, Mosquitoes, Host preference, Vector surveillance, Ethiopia

## Abstract

**Background:**

Arboviruses continue to threaten global health because of their rapid geographical expansion and significant disease burden. Of the over 500 recognized arboviruses, approximately 150 affect humans, and around 50 affect domestic animals and wildlife. The spread and impact of these viruses have increased significantly over the past three decades, driven by the proliferation of their vectors and the rise of global trade and travel.

**Methods:**

In this study, we used molecular methods to characterize mosquito species diversity and host feeding preferences across Ethiopia’s Great Rift Valley. Mosquitoes were collected from diverse habitats in the Great Rift Valley of Ethiopia using Centers for Disease Control and Prevention (CDC) light traps, BG-Sentinel traps, and hand aspirators. The area was chosen for its high vector diversity, suitable breeding habitats, and the epidemiological importance of arboviruses. Morphological identification was conducted, and 204 blood-fed mosquitoes were selected. Genomic DNA was extracted, followed by polymerase chain reaction (PCR) amplification targeting the *COI* gene. Blood meal analysis was performed using vertebrate-specific primers targeting the *12S* rRNA gene. Mosquito species identification, genetic diversity analysis, and phylogenetic analyses were conducted.

**Results:**

Of 6601 collected mosquitoes, 4977 were identified morphologically, comprising 399 *Aedes*, 2861 *Culex*, 1841 *Anopheles*, and 275 *Mansonia* species. *COI* DNA barcode analysis identified 142 mosquito specimens belonging to 16 species, with *Anopheles coustani*, *Cx. tritaeniorhynchus*, *Cx. pipiens* complex, *Mansonia africana*, and *Ma. uniformis* being the predominant species. Blood meal analysis (*n* = 71 successful amplifications) revealed a primary reliance on humans and cattle. *Cx. pipiens* complex showed a strong anthropophilic tendency, while *Cx. tritaeniorhynchus* and *Ma. uniformis* exhibited broader host ranges. Genetic diversity indices showed significant Fu’s *F*_s_ statistics for *Cx. pipiens* complex, *Cx. tritaeniorhynchus*, *Ma. africana*, and *Ma. uniformis*.

**Conclusions:**

This study offers valuable preliminary insights into the diversity of mosquito species, genetic variation, and host-feeding preferences within the Ethiopian Rift Valley. The findings emphasize the potential of molecular techniques to enhance traditional entomological methods and improve the accuracy of mosquito identification. While the study is limited in both geographic and temporal scope, it highlights mosquito species of medical and veterinary significance and suggests implications for arboviral disease surveillance.

**Graphical Abstract:**

**Figure:**
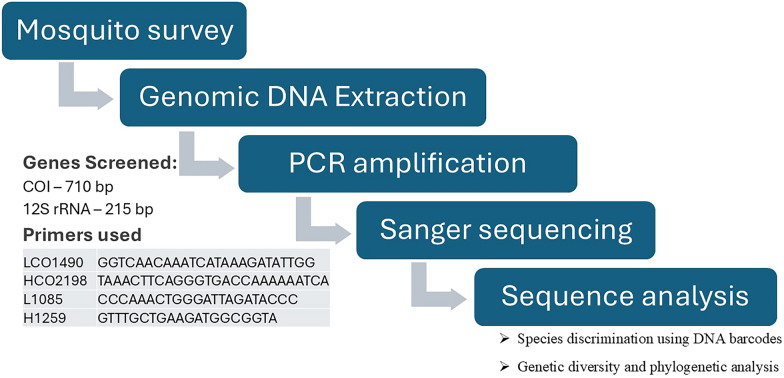

## Background

Arboviruses, viruses transmitted primarily by arthropod vectors such as mosquitoes, represent a significant and growing threat owing to their rapid geographical expansion and substantial disease burden [1]. Of the over 500 known arboviruses, approximately 150 can cause human diseases, and around 50 affect domestic animals and wildlife [2, 3]. Over the past three decades, the distribution and impact of arboviruses have surged [4, 5], fueled by the widespread proliferation of their vectors alongside increases in global trade and travel [6]. This trend underscores the urgent need for innovative and effective surveillance strategies.

Mosquitoes (Diptera: Culicidae) play a central role in arbovirus transmission, significantly contributing to the burden of diseases such as dengue, Zika, yellow fever, chikungunya, West Nile virus (WNV), and Rift Valley fever virus (RVFV) [4]. Currently, 3727 mosquito species are formally recognized within the family *Culicidae* [7]. Out of 215 countries/territories worldwide considered suitable for at least one of these vectors, 146 have reported at least one instance of arboviral disease transmission [5]. In Ethiopia, where dengue virus (DENV) and yellow fever virus (YFV) are endemic, and RVFV poses a high risk, the demand for robust and tailored surveillance methods is high [5, 8].

Molecular techniques have revolutionized the field of vector surveillance by enabling more precise identification of mosquito species and their blood meal preferences [9]. Accurate identification of vectors is essential for developing effective strategies to manage arboviral diseases [10]. Traditionally, morphological characterization has been the primary method for identifying mosquitoes. While this approach is still widely used, it has significant limitations: It requires considerable expertise, is time-consuming, and struggles to distinguish morphologically similar species [11–13]. Despite these constraints, previous studies have primarily relied on morphological features to survey mosquito populations in Ethiopia [14–16]. Recently, there has been a growing need to shift toward DNA-based methods, which offer greater precision [13, 17]. These molecular approaches often utilize mitochondrial genes because of their high efficiency in distinguishing mosquito species and revealing phylogenetic relationships [18, 19].

Recent studies in Ethiopia illustrate the value of molecular surveillance. A multisite survey that combined molecular and morphological methods improved *Anopheles* species identification and revealed diverse local vector behaviors relevant to control strategies [20]. Similarly, population-genetic analyses of *Anopheles stephensi* uncovered significant genetic diversity and geographic structuring, emphasizing the utility of DNA barcoding and host profiling for understanding species spread and epidemiological risk [21]. However, most molecular efforts to date have focused on *Anopheles*, while arboviral vectors such as *Aedes* and *Culex* remain understudied.

Beyond mosquito species identification, molecular techniques enable the analysis of mosquito blood meals, providing critical insights into host preferences and potential pathways of virus transmission [22]. These insights help public health authorities to design and implement effective arboviral disease control strategies. Molecular markers, notably mitochondrial genomes, are commonly used to determine host range preferences precisely [23, 24].

Despite their advantages, molecular techniques remain underutilized in many low- and middle-income countries, including Ethiopia. Challenges such as the lack of updated morphological keys and limited expertise in medical and veterinary entomology further complicate accurate vector identification. These limitations likely contribute to the scarcity of molecular-based vector surveillance studies across African countries.

Therefore, this study aimed to characterize mosquito species diversity and host-feeding patterns in Ethiopia’s Rift Valley using molecular techniques, providing essential baseline data on the composition and feeding behavior of Ethiopian mosquito populations.

## Methods

### Study area

**Fig. 1:**
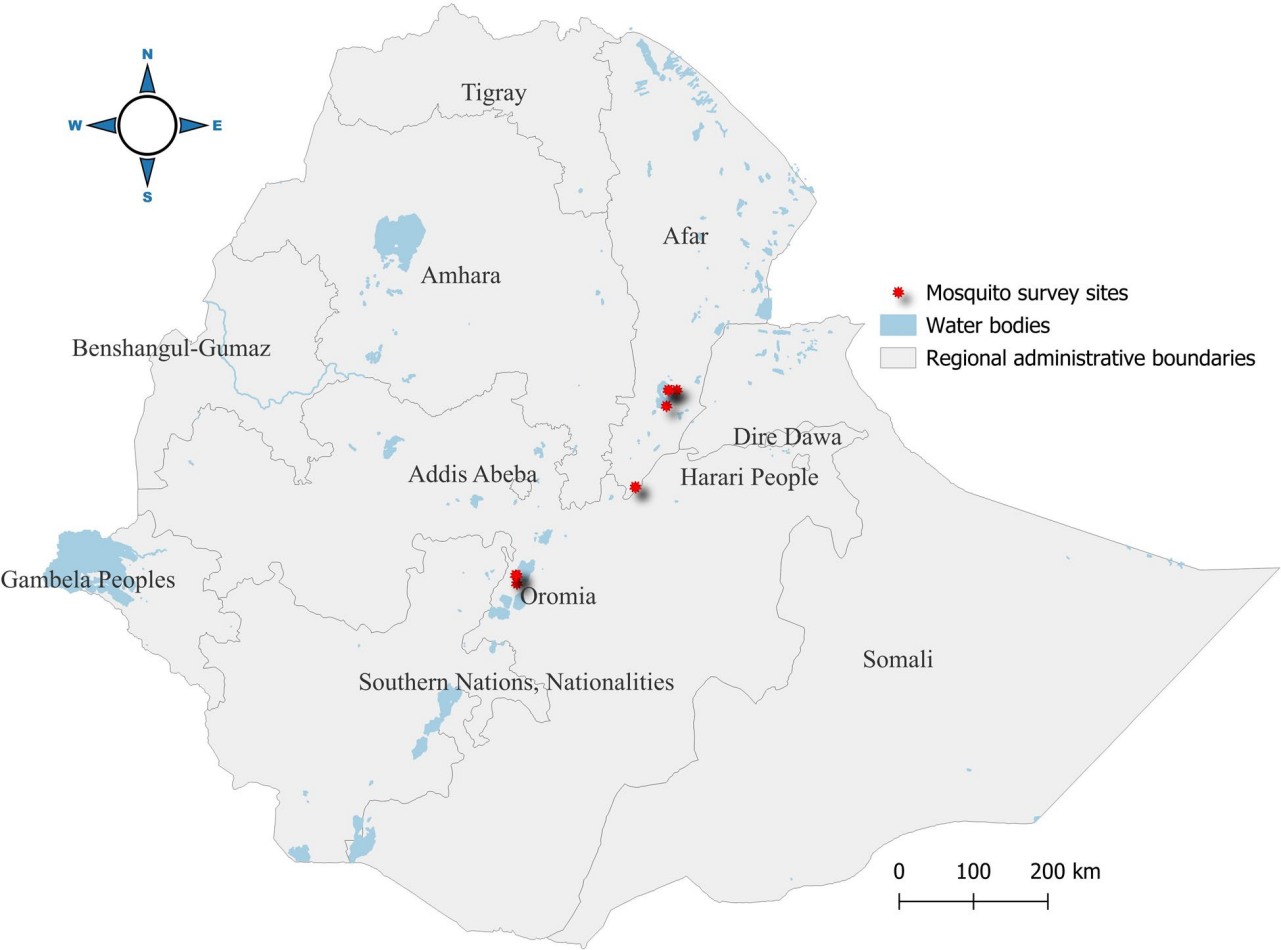
Map of the study area. The areas indicated by red dots are sampling points, and bar graphs show mosquito species collection per site

The present study was conducted in Ethiopia’s Great Rift Valley (Fig. 1). The area was chosen for its high vector diversity, suitable breeding habitats, and the epidemiological importance of arboviruses. The study area extends from Batu (7°55′51″N, 38°42′58″E) to Gawane (10°9′5″N, 40°38′43″E). Collection sites were strategically chosen to encompass a range of habitats, including urban, peri-urban, and rural environments. The selected study areas are believed to support oviposition and the development of mosquito larvae year-round, as these areas have large permanent water bodies and warmer temperatures.

### Mosquito collection and identification

Mosquitoes were collected using Centers for Disease Control and Prevention (CDC) light traps, BG-Sentinel traps, and hand aspirators. Collections were carried out in August 2023 during the main rainy season, which corresponds to peak mosquito abundance. The restriction to 1 month was owing to logistical and resource constraints. In total, 24 traps were deployed near potential mosquito oviposition and feeding sites, including indoor and outdoor areas near water bodies, animal enclosures, and fields with dense human and livestock populations in four sites (Awash 7, Batu, Gelealo, and Gawane).

Mosquitoes have diverse feeding behaviors [25]. To accommodate the different feeding times of the mosquitoes, the traps were placed from 16:00 to 18:00 and collected between 7:00 and 9:00 the following day. The collection cups were briefly frozen at −20 °C for 15 min to immobilize the mosquitoes. Mosquitoes were morphologically identified to the genus level using dichotomous keys from the Walter Reed Biosystematics Unit (WRBU) [26, 27]. Out of the identified mosquitoes, 204 blood-fed specimens were further processed for DNA barcoding and blood meal analysis.

Species names adhere to the nomenclature used in the online resource (www.mosquito-taxonomic-inventory.info) [7]. For comprehensive specimen data, including collection details and GenBank accession numbers, refer to the “Availability of data and materials” section.

### DNA extraction and amplification

Genomic DNA from the selected mosquitoes was extracted using DNeasy Blood & Tissue and MACHEREY–NAGEL NucleoSpin DNA/RNA extraction kits following the manufacturer’s instructions with slight modifications. The tissues were macerated using an electric homogenizer (Precellys Evolution Touch) in a 1.5 ml microcentrifuge tube containing sterile beads. Amplification of the 710 base pair (bp) fragment flanking the *COI* gene was performed using the primer set LCO1490 (5′-GGTCAACAAATCATAAAGATATTGG-3′) and HCO2198 (5′-TAAACTTCAGGGTGACCAAAAAATCA-3′) using polymerase chain reaction (PCR) [28]. PCR reactions were performed in a final volume of 25 μl, containing 2.5 μl of 10× buffer, 0.5 μl of 10 mM dNTP mix, 0.5 μl each of 10 μM forward and reverse primers, 0.1 μl of Taq DNA polymerase (5.0 U/μl), 1 μl of the extracted DNA template, and 19.9 μl of double-distilled (dd)H_2_O. PCR conditions were initial denaturation at 94 °C for 1 min, followed by 35 cycles of denaturation at 94 °C for 40 s, annealing at 49 °C for 40 s, extension at 72 °C for 1 min, and a final extension at 72 °C for 5 min. PCR products were examined on a 1.5% agarose gel stained with Midori Green. Amplified PCR products were purified and sequenced using Eurofins’ Sanger sequencing technique. DNA from *Ae. aegypti*, obtained from the Rega Institute, KU Leuven, served as the positive control, and ddH_2_O was used as a negative control.

### Molecular identification of blood meals

During field sampling, the abdomens of selected blood-fed mosquitoes were squeezed onto a Flinders Technology Associates (FTA) card and stored at room temperature until extraction. Genomic DNA was extracted from the FTA card using a DNeasy Blood & Tissue kit. Subsequently, PCR was performed to amplify specific regions of vertebrate mitochondrial DNA. Universal vertebrate-specific primers (L1085: forward 5′- CCCAAACTGGGATTAGATACCC-3′ and H1259: reverse 5′- GTTTGCTGAAGATGGCGGTA-3′) were used to amplify a 215-bp segment of the host *12S* rRNA loci mitochondrial genome [24]. PCR conditions were initial denaturation at 95 °C for 1 min, followed by 35 cycles of denaturation at 95 °C for 30 s, annealing at 50 °C for 40 s, extension at 72 °C for 1 min, and a final extension at 72 °C for 5 min. PCR products were examined on 2% agarose gels stained with Midori Green. Amplified PCR products were purified and sequenced using Eurofins’ Sanger sequencing technique. The identity of the blood meals was determined by comparing the sequences in GenBank and identifying the closest matches at the species level of vertebrate hosts.

### Sequence and data analysis

#### Mosquito species discrimination using DNA barcodes.

Chromatogram inspection and assembly of forward and reverse sequences and pairwise alignment were performed using BioEdit Sequence Alignment Editor version 7.2.5. Raw sequences were first examined for base-calling accuracy; low-quality ends were trimmed, and sequences with extensive ambiguous bases, stop codons, or poor chromatogram quality were excluded from downstream analysis. Consensus sequences were generated from forward and reverse reads following manual editing of ambiguous sites. Species-level identification was performed by analyzing *COI* sequences using the BOLD Systems identification tool [29] and National Center for Biotechnology Information (NCBI) Basic Local Alignment Search Tool (BLAST) [30]. The approach relied on higher similarity in the BOLD Systems identification tool and homology search results from GenBank sequences. In the event of a discrepancy between the two methods, an additional step was taken by conducting an NCBI BLAST using voucher sequences. If the discrepancy persists, the result of the BOLD Identification System was upheld because it has been shown to outperform GenBank/NCBI, as noted by Baena-Bejarano et al. [31].

### Genetic diversity and phylogenetic analysis

The barcode gap analysis (BGA) and Kimura 2-parameter (K2P) genetic distance calculations were performed using BOLD version 4 [29]. The number of polymorphic sites, average number of pairwise nucleotide differences, nucleotide diversity, number of haplotypes, haplotype diversity, as well as neutrality test (Fu’s *F*_s_ statistics) were analyzed using the DNA Sequences Polymorphism software (DnaSP version 6.12.03) [32].

Phylogenetic analysis was conducted utilizing Geneious Prime software. *COI* gene sequences of mosquito species were downloaded from GenBank and BOLD Systems, focusing on sequences originating from neighboring African countries and possessing a length exceeding 600 base pairs. Multiple alignments were performed using MAFFT. Subsequently, a phylogenetic tree was generated employing the Tamura–Nei genetic distance model and the neighbor-joining tree-building method via Geneious Tree Builder. Bootstrap coefficients were computed for 1000 replicates, and only those exceeding a support value of 75% were integrated into the final representation of the tree.

## Results

A total of 6601 mosquitoes were collected, of which 4977 were identified to the genus level morphologically. Specifically, the breakdown comprised 399 *Aedes*, 2861 *Culex*, 1841 *Anopheles*, and 275 *Mansonia* species. Table 1 summarizes the results of mosquito trapping across various sites, detailing the number of traps deployed, total catches, and species identified. In total, 24 traps were set across four sites: Awash 7, Batu, Gelealo, and Gawane. The number of catches varied significantly by site, with Awash yielding 1018 mosquitoes, Batu Town 446, Gelealo 4080, and Gawane 1057. The species diversity was also notable across the sites. Notably, the highest mosquito catch was recorded in Gelealo, in the Afar region, with an average of 2040 mosquitoes per trap. Numerous mosquito specimens were unidentifiable owing to severe damage and morphological distortion within the trap cups. This damage was likely linked to the high catch rate, as mosquitoes tend to crush one another while attempting to escape from the trap. In areas where high mosquito collections are expected, increasing the frequency of trap collections can help reduce overcrowding, thereby minimizing damage and improving sample quality.

**Table 1:** Number of traps deployed per site, number of catches, and species identified per site

Sites	Number of traps deployed	Number of catches			Total number of engorged mosquitoes processed	Species identified
		Indoor	Outdoor	Total		
Awash 7	4	767 (2)	251 (2)	1018	71	*An. Coustani* (1), *Cx. pipiens* complex (38), *Cx. Quinquefasciatus *(1), *Culex* spp. (8)
Batu	12	55 (1)	69 (11)	446	29	*An. coustani* (4), *An. gambiae* (1), *An. pharoensis* (1), *Coquillettidia microannulata* (1), *Cx. neavei* (2), *Cx. pipiens* complex (9), *Cx. rima* (1), *Cx. tenagius* (3), *Cx. tritaeniorhynchus* (2), *Ma. africana* (1), and *Ma. uniformis* (1)
Gelealo	2	480 (1)	3600 (1)	4080	10	*An. Coustani* (1), *Culex* spp. (1)
Gawane	6	186 (1)	871 (5)	1057	94	*Aedes aegypti* (1), *Ae. mcintoshi* (1), *Ae. natronius* (1), *An. coustani* (2), *Cx. neavei* (5), *Cx. pipiens* complex (14), *Cx. quinquefasciatus* (4), *Cx. univittatus* (2), *Cx. tenagius* (1), *Cx. tritaeniorhynchus* (22), *Culex* spp. (4), *Ma. africana* (9), *Ma. uniformis* (13)

### *COI*-based DNA barcodes and phylogenetics

The analysis of 204 mosquito *COI* DNA barcode sequences confirmed the identification of 142 mosquito specimens belonging to 16 species: *Ae. aegypti* (*n* = 1), *Ae. mcintoshi* (*n* = 1), *Ae. natronius* (*n* = 1), *An. coustani* (*n* = 8), *An. gambiae* (*n* = 1), *An. pharoensis* (*n* = 1), *Cq. microannulata* (*n* = 1), *Cx. neavei* (*n* = 7), *Cx. pipiens* complex (*n* = 61), *Cx. quinquefasciatus* (*n *= 5), *Cx. rima* (*n* = 1), *Cx. tenagius* (*n* = 4), *Cx. tritaeniorhynchus* (*n* = 24), *Cx. univittatus* (*n* = 2), *Ma. africana* (*n* = 10), and *Ma. uniformis* (*n* = 14). However, 52 samples that were initially identified at the genus level, *Aedes* sp. (1), *Anopheles* sp. (1), *Culex* sp. (49), and *Mansonia* sp. (1), could not be further analyzed owing to low sequence similarity in the BOLD Systems identification tool and homology searches in GenBank; 15 of these samples exhibited poor chromatogram quality and contained stop codons within their sequences. In addition, ten samples were unable to undergo amplification. It is noteworthy that *Cq. microannulata*, identified using molecular techniques, could not be identified even at the genus level using morphological identification.

Of the 155 mosquito sequences submitted to the BOLD system, 143 have been associated with a Barcode Index Number (BIN). This system allocates a distinctive global identifier to each sequence cluster. While specimens allocated to different BINs typically correspond to distinct species, the BIN system facilitates data organization for records lacking a formal taxonomic assignment.

The BIN system has categorized the 15 species into 16 distinct BINs. *Cx. pipiens* complex and *Cx. quinquefasciatus* have been categorized into a BIN (BOLD: AAA4751), with over 8784 members. *Cx. tritaeniorhynchus* has been divided into two distinct BINs, with the majority (96%) in BOLD: AAE3201, which has over 3291 members. *An. coustani* was also categorized into two distinct BINs, with most members (87.5%) classified into BOLD: AAN9442, with over 680 members. Meanwhile, *Cx. tenagius*, *Cx. neavei*, *Cx. univittatus*, *Ma. africana*, and *Ma. uniformis* were each classified into distinct BINs.

The BGA analysis shows a notable distinction between intraspecific and interspecific (nearest neighbor) distances across most species, with apparent exceptions observed, particularly between *Cx. pipiens* complex and *Cx. quinquefasciatus*, as well as between *Cx. neavei* and *Cx. rima*. Notably, for *An. coustani*, *Cx. tenagius*, *Cx. tritaeniorhynchus*, *Cx. univittatus*, *Ma. africana*, and *Ma. uniformis*, the distance to the nearest neighbor surpassed the maximum intraspecific distance.

Intraspecific distances could not be determined for six species, namely *Ae. aegypti*, *Ae. mcintoshi*, *Ae. natronius*, *An. gambiae*, *An. pharoensis*, and *Cq. microannulata*, as each was represented by a single specimen. Nonetheless, all these species exhibited nearest-neighbor distances exceeding 7%.

Sequence divergence increased as the taxonomic rank rose. Intraspecific divergences ranged from 0.0% to 27.2%, averaging 3.7%, whereas divergences within species of a genus ranged from 0.2% to 70%, averaging 10.9% (Table 2). However, upon excluding species where interspecific distances surpass intraspecific distances, notably the *Cx. pipiens* complex, *Cx. neavei*, and *Cx. rima*, there was no overlap, indicating clear distinctions among species.

**Table 2:** K2P sequence divergence at the *COI* barcode region among the mosquito species with greater than two specimens, among the four genera with two or more species, and in the family Culicidae

Distance class	*n*	Taxa	Comparisons	Minimum (%)	Mean (%)	Maximum (%)
All sequences						
Within species	148	10	2368	0	3.7	27.2
Within genus	154	4	4742	0.2	10.9	70
Selected species^*^						
Within species	62	6	447	0	1.7	7.4
Within genus	54	2	292	7.9	10.6	15.9

^*^Selected sequences: *An. coustani*, *Cx. tenagius*, *Cx. tritaeniorhynchus*, *Cx. univittatus*, *Ma. africana*, and *Ma. uniformis*

Genetic diversity indices and results of neutrality tests are presented in Table 3. The average number of pairwise nucleotide differences (*k*), nucleotide diversity (*π*), and haplotype diversity (Hd) varied for the species. Fu’s *F*_s_ statistics were significant for *Cx. pipiens* complex, *Cx. tritaeniorhynchus*, *Ma. africana*, and *Ma. uniformis*.

**Table 3:** Genetic diversity indices and neutrality test in the *COI* gene for the most common mosquito species from Ethiopia

Mosquito species	*n*	*s*	*h*	Hd ± SD	*k*	*π*	Fu’s *F*_s_ statistic
*An. coustani*	8	16	5	0.79 ± 0.15	5	0.011	0.79
*Cx. neavei*	7	0	1	0	0	0.000	–
*Cx. pipiens* complex	61	30	30	0.88 ± 0.04	4	0.037	**−19.50**
*Cx. quinquefasciatus*	5	52	5	1 ± 0.13	25.8	0.043	0.81
*Cx. tenagius*	4	1	2	0.50 ± 0.27	0.5	0.001	0.17
*Cx. tritaeniorhynchus*	24	29	16	0.90 ± 0.06	4.9	0.010	**−6.34**
*Cx. univittatus*	2	4	2	1 ± 0.50	4	0.006	NA
*Ma. africana*	10	13	9	0.98 ± 0.05	3.9	0.006	**−4.51**
*Ma. uniformis*	14	14	12	0.97 ± 0.04	2.6	0.005	**−9.49**

Fu’s *F*_s_: A negative value of *F*_s_ is evidence of an excess number of alleles, as expected from a recent population expansion or genetic hitchhiking.

The bolded Fu’s *F*_s_ statistic values are statistically significant. Species represented by less than three specimens or sequences with less than 500 bp were not included in the analyses.

*NA* not applicable (at least four sequences needed), *n* number of sequences, *s* number of polymorphic sites, *k* average number of pairwise nucleotide differences, *π* nucleotide diversity, *h* number of haplotypes, *Hd* haplotype diversity.

**Fig. 2:**
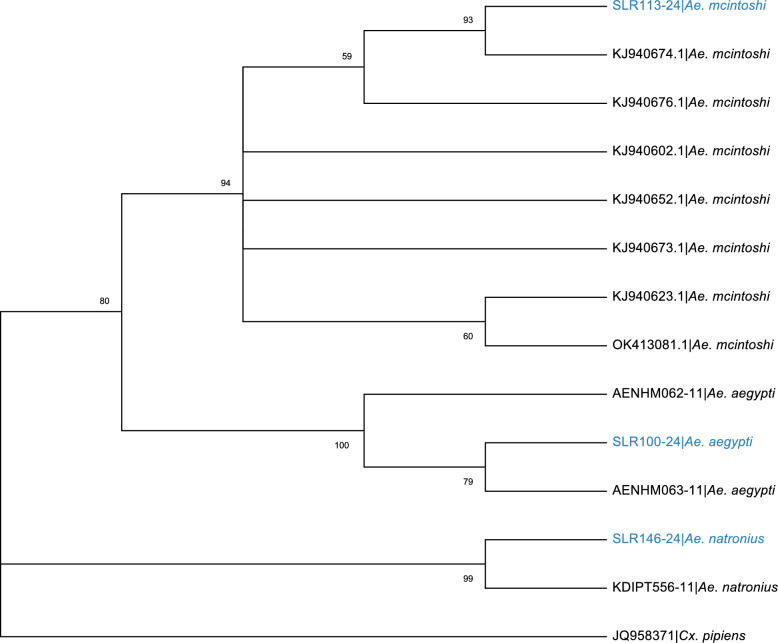
Phylogenetic tree of *Aedes* mosquito species based on *COI* gene. The tree includes sequences from *Ae. mcintoshi*, *Ae. natronius*, and *Ae. aegypti*, with *An. coustani* as an outgroup. Bootstrap support values are shown at the nodes. Our sequences are highlighted in blue

The phylogenetic analysis of the *Aedes* genus reveals that *Ae. mcintoshi* forms a distinct group but also exhibits close relationships with *Ae. natronius*. In contrast, *Ae. aegypti* forms another distinct and highly supported group (Fig. 2).

**Fig. 3:**
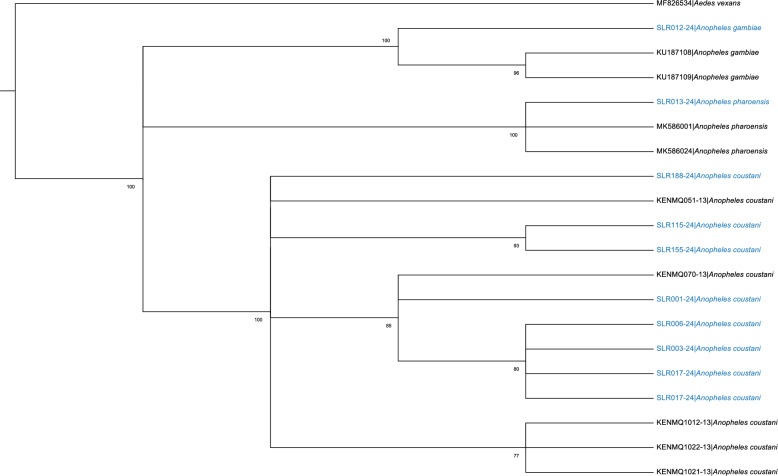
Phylogenetic tree of *Anopheles* species based on *COI* gene. The tree includes sequences from *An. pharoensis*, *An. gambiae*, and *An. coustani*, with *Ae. vexans* as an outgroup. Bootstrap support values are shown at the nodes. Our sequences are shown in blue

Figure 3 shows the evolutionary relationships among the three *Anopheles* species identified in this study. The phylogenetic tree shows that *An. coustani*, *An. gambiae*, and *An. pharoensis* forms distinct and strongly supported groups within their respective clades.

**Fig. 4:**
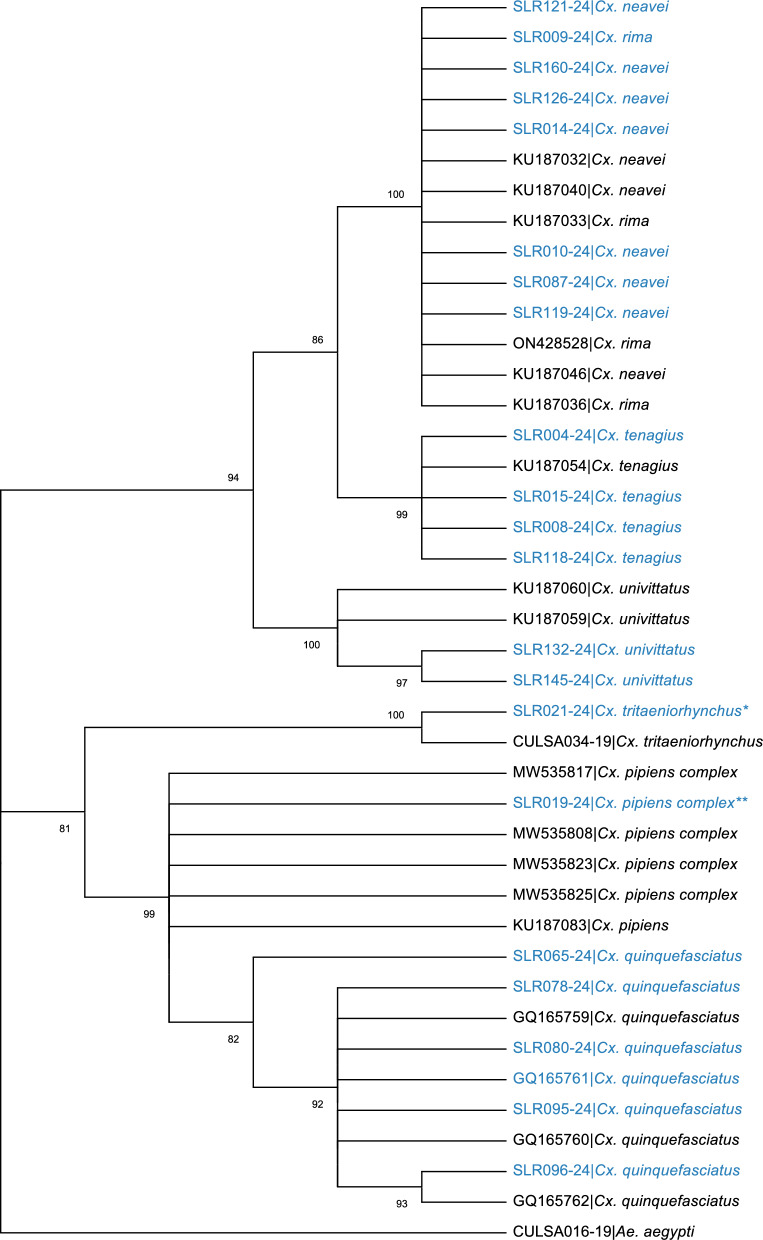
Phylogenetic tree of *Culex* species based on *COI* gene. The tree includes sequences from different *Culex* species, with *Ae. aegypti* as an outgroup. Bootstrap support values are shown at the nodes. Our sequences are shown in blue. Key (*) 23 *Cx. tritaeniorhynchus* sequences belonging to the same haplotype merged and (**) 51 *Cx. pipiens* complex sequences belonging to the same haplotype merged

Figure 4 shows the phylogenetic analysis of the species in the genus *Culex*. The phylogenetic tree depicts distinct and strongly supported groups for *Cx. tritaeniorhynchus*, *Cx. univittatus*, and *Cx. tenagius*. However, the *Cx. pipiens* complex and *Cx. quinquefasciatus* are grouped with high bootstrap support (99), indicating a strong genetic relationship. *Cx. neavei* and *Cx. rima* are also grouped together with high bootstrap support (100). These results align with the BGA analysis, showing the same relationships.

**Fig. 5:**
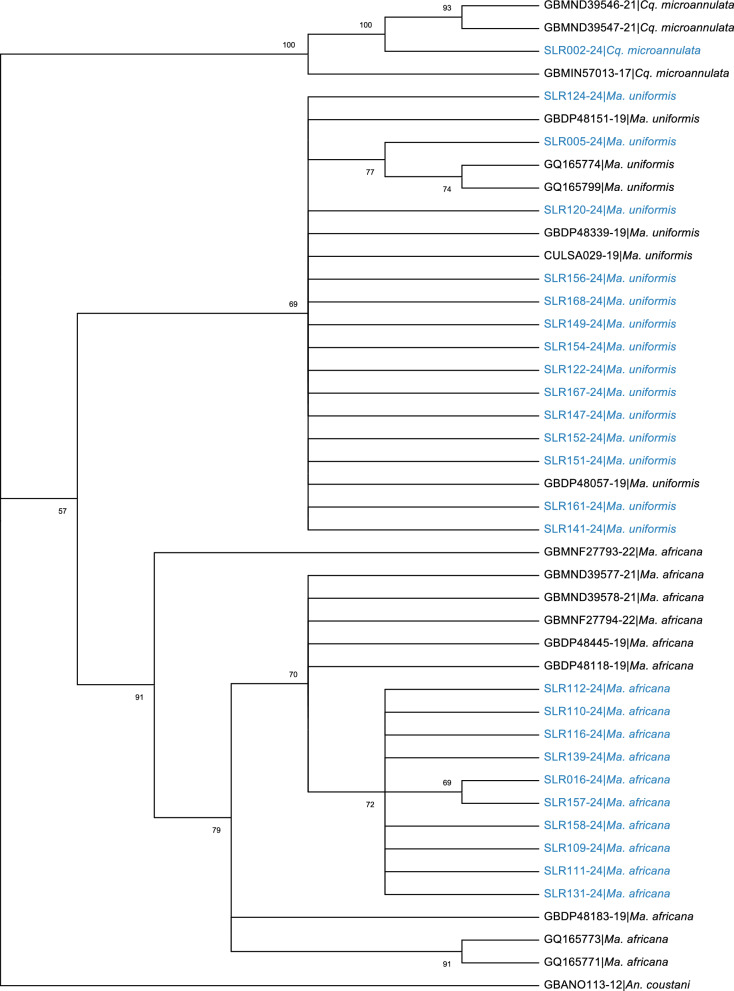
Phylogenetic tree of species in the tribe Mansoniini based on *COI* gene. The tree includes sequences from *Ma. africana*, *Ma. uniformis*, and *Cq. microannulata*, with *An. coustani* as an outgroup. Bootstrap support values are shown at the nodes. Our sequences are shown in blue

As shown in Fig. 5, *Ma. africana*, *Ma. uniformis*, and *Cq. microannulata* form distinct and strongly supported groups within their respective clades. The tree splits into two major clades after the outgroup. One clade contains *Ma. africana* and *Ma. uniformis*, while the other contains *Cq. microannulata*. These species belong to the same tribe (Mansoniini), but *Cq. microannulata* belongs to the genus *Coquillettidia*, whereas *Ma. africana* and *Ma. uniformis* belong to the genus *Mansonia*.

### Blood meal analysis

Table 4 summarizes the host-derived blood meals identified from various mosquito species on the basis of the host *12S* rRNA gene. Of the 204 specimens collected, 142 were successfully barcoded and subsequently analyzed for blood meals. Host DNA was successfully amplified in 71 samples, whereas amplification failed in the remaining samples owing to poor DNA quality or low chromatogram resolution. The analysis revealed a predominant reliance on human and cattle hosts. Notably, *Cx. tritaeniorhynchus* and *Ma. uniformis* exhibited diverse host preferences, whereas *Cx. pipiens* complex demonstrated a pronounced tendency toward human feeding. Amplification of host-derived blood meals was unsuccessful for *Ae. mcintoshi*, *An. gambiae*, *An. pharoensis*, and *Cq. microannulata*, all of which are represented by a single specimen.

**Table 4:** Summary of host-derived blood meals in identified mosquitoes

Mosquito species	Number analyzed	Number amplified (%)^*^	Host
*Ae. aegypti*	1	1 (100)	Human
*Ae. mcintoshi*	1	–	
*Ae. natronius*	1	1 (100)	Cattle
*An. coustani*	8	2 (25)	Cattle
*An. gambiae*	1	–	
*An. pharoensis*	1	–	
*Cq. microannulata*	1	–	
*Cx. neavei*	7	4 (57)	Cattle
*Cx. pipiens* complex	61	32 (52.5)	Human (31), chicken (1)
*Cx. quinquefasciatus*	5	1 (20)	Human
*Cx. rima*	1	1 (100)	Human
*Cx. tenagius*	4	2 (50)	Cattle (1), sheep (1)
*Cx. tritaeniorhynchus*	24	15 (62.5)	Human (1), donkey (2), cattle (8), sheep (1), goat (1), rat (1), bat (1)
*Cx. univittatus*	2	1 (50)	Role-rat
*Ma. africana*	10	4 (40)	Cattle (2), goat (2)
*Ma. uniformis*	14	7 (50)	Cattle (4), donkey (1), rodent (1), sheep (1)

^*^Percentage calculated as (the number of the blood-fed specimens analyzed/the number of blood-fed specimens with amplification host) × 100

## Discussion

Ethiopia faces serious public health challenges from arboviral diseases such as dengue, Chikungunya, RVFV, and yellow fever virus [5, 8]. Previous research in the country has mainly focused on malaria vectors, particularly species in the *Anophelinae* subfamily [14, 20, 33–35]. However, species from the *Culicinae* subfamily are also significant arboviral vectors [36]. The present study, which utilized *COI*-based DNA barcoding and blood meal analysis, provides one of the first molecular characterizations of mosquito species in the Great Rift Valley of Ethiopia, thereby contributing to the limited genetic data available on the country’s arbovirus vectors. While traditional surveillance relies on morphological identification, which is error-prone for species complexes [11, 12], we utilized *COI* barcoding to improve accuracy. However, it is important to note that the use of a single mitochondrial marker (*COI*) and Sanger sequencing, while effective for initial species identification, has inherent limitations for resolving recent hybridization or complex evolutionary histories. With this methodological context in mind, our findings offer valuable initial insights into the species composition and feeding behaviors of mosquitoes in this understudied region.

Traditional mosquito surveillance methods typically rely on morphological identification, which can be challenging and error-prone, especially when dealing with closely related species [11, 12]. This study used a molecular approach to address these limitations, offer more accurate identification, and uncover cryptic species that conventional methods might overlook. DNA barcoding enables the precise identification of mosquito species, including morphologically similar ones [11, 12]. Moreover, by analyzing the blood meals of blood-fed mosquitoes, this study reveals the host preferences of various mosquito species. This information is crucial for identifying potential reservoirs and amplifying hosts of arboviruses, thereby facilitating targeted control strategies [22].

The distribution and abundance of mosquito species varied significantly across the study sites. Notably, Gelealo, in the Afar region, recorded the highest number of mosquitoes, with an average of 2040 per trap, predominantly *Culex* spp, which could be potential hotspots for arboviral transmission that necessitate targeted vector control measures. In contrast, Batu Town had lower catches but a more diverse mosquito population, including *Anopheles*, *Coquillettidia*, and *Mansonia* species. These results are in agreement with the previous report by Jaleta et al. [16].

Close genetic relationships were observed between certain species pairs, such as *Cx. neavei* and *Cx. rima*, suggesting potential evolutionary links that warrant further investigation. However, the close genetic relationship between *Cx. pipiens* complex and *Cx. quinquefasciatus* is expected since *Cx. quinquefasciatus* is a member of the *Cx. pipiens* complex group. The *Cx. pipiens* complex species are difficult to distinguish owing to their morphological similarity and close genetic relationship, and they are key vectors in the transmission of various pathogens. This complex includes *Cx. pipiens pipiens*, which has two forms, pipiens and molestus, *Cx. pipiens pallens*, *Cx. quinquefasciatus*, *Cx. australicus*, and *Cx. globocoxitus*. Among these, *Cx. pipiens pipiens* and *Cx. quinquefasciatus* are the most widespread species [37, 38].

The observed high haplotype and nucleotide diversity within the *Cx. pipiens* complex indicates substantial genetic variation within this population. This has direct implications for vector control in Ethiopia. Genetically diverse populations may have a higher capacity to adapt to environmental changes, such as urbanization in the Rift Valley, and potentially develop resistance to commonly used insecticides. The significant Fu’s *F*_s_ statistic further suggests recent population expansion or genetic hitchhiking [39], potentially linked to increasing urbanization or changing climatic patterns in the region, which could be expanding favorable habitats for this vector.

Our method, relying on *COI*, was able to identify *Cx. quinquefasciatus* but could not resolve the remaining members of the complex, highlighting a key limitation of a single-marker approach. The pattern we observed is consistent with potential hybridization, a known phenomenon in this complex. This matters for transmission because hybrids often exhibit “bridge” vector behavior, feeding on both birds and humans [38, 40]. The presence of such hybrids in the Ethiopian Rift Valley would significantly elevate the risk of zoonotic virus spillover. Therefore, detailed molecular studies incorporating additional markers such as the Internal Transcribed Spacer 2 (ITS2) and *16S* ribosomal RNA (*16S* rRNA) are needed to accurately identify species within this group and understand the extent of hybridization. Recent advances also highlight the potential of multi-locus amplicon panels and computational approaches to overcome the limitations of *COI* alone, particularly in resolving cryptic species complexes [41]. Adopting such complementary methods could greatly enhance the resolution and reliability of mosquito species identification in future studies.

Beyond the *Cx. pipiens* complex, the analysis of genetic diversity indices provided valuable insights into the population structures of other mosquito species. A significant Fu’s *F*_s_ statistic was observed for *Cx. tritaeniorhynchus*, *Ma. uniformis*, and *Ma. africana*, indicating recent population expansions or genetic hitchhiking, potentially in response to environmental changes or selective pressures. While there are no similar studies specifically for *Ma. uniformis* and *Ma. africana*, a study from Türkiye [42] indicated high genetic diversity for *Cx. tritaeniorhynchus*, supporting the presence of substantial genetic variability in this species.

In this study, *An. coustani*, *Cx. pipiens* complex, *Cx. tritaeniorhynchus*, and *Ma. uniformis* emerged as the predominant mosquito species. A recent review by Adugna et al. [34] highlighted *An. coustani* as a widespread *Anopheles* species in Ethiopia alongside *An. arabiensis* and *An. pharoensis*. Known as a malaria vector in Kenya [43] and Madagascar [44], *An. coustani* is suspected to play a role in malaria transmission in Ethiopia as well, where malaria remains a leading cause of illness and mortality [45]. In addition to malaria, *An. coustani* is implicated in the transmission of RVFV and Zika virus (ZIKV) [46, 47].

The *Cx. pipiens* complex is primarily associated with the transmission of WNV [48]; however, it is also known to transmit Japanese encephalitis virus (JEV), St. Louis encephalitis virus, Usutu virus, and Sindbis virus [37, 49]. Similarly, *Cx. tritaeniorhynchus* is a significant vector for several arboviruses, including JEV, RVFV, WNV, and Tembusu viruses, affecting humans and animals [42]. *Culex tritaeniorhynchus* is the principal vector of JEV, and pigs are its primary amplifying hosts [50]. In Ethiopia, the current risk of JEV transmission is considered low owing to the limited extent of swine farming. However, if swine farming expands in the future, the combination of a widespread competent vector and an amplifying host could increase the risk of JEV emergence.

*Ma. uniformis* is another important species identified in the Great Rift Valley of Ethiopia [16]. It plays a crucial role in transmitting various pathogens and parasites responsible for diseases of primary medical and veterinary importance. Notably, *Ma. uniformis* is a competent vector for several emerging and re-emerging arboviruses, such as Chikungunya virus (CHIKV), ZIKV, JEV, RVFV, and WNV. Beyond viral infections, it is also recognized as a competent vector for filariasis, avian malaria, and monkey malaria [51].

*Ae. aegypti* is recognized as one of the most cosmopolitan mosquito species and serves as a primary vector for several emerging and reemerging arboviruses, including DENV, CHIKV, ZIKV, and YFV [5]. In contrast, *Ae. mcintoshi* is identified as the predominant vector of RVFV in East Africa. The abundance and feeding patterns of *Ae. mcintoshi* in flood-prone areas significantly contribute to its role in RVF disease outbreaks in East Africa [52]. This study reported both species as singletons, likely owing to the sampling constraints.

A review conducted by Adugna et al. [34] reported 35 mosquito species belonging to the *Anophelinae* subfamily, while a study by Jaleta et al. [16] identified more than 10 mosquito species within the *Culicinae* subfamily. Therefore, the mosquito species diversity documented in the present study may not fully capture the actual diversity present in the country. This limitation may be attributed to several factors, including logistical constraints such as the absence of CO_2_ attractants, as well as seasonal and geographic restrictions in sampling. Notably, studies have demonstrated that CO_2_-baited traps can increase mosquito capture rates by several-fold compared with unbaited traps [53]. Seasonal variations play a role in the occurrence and abundance of mosquitoes [54]. Our survey was conducted during the main rainy season, which means that our findings may not fully reflect the situation in other seasons. Furthermore, given Ethiopia’s ecological heterogeneity, mosquito diversity is likely to vary across different eco-epidemiological zones. Consequently, nationwide surveys covering multiple regions and seasons will be essential before broader generalizations can be made about the country’s mosquito fauna and their epidemiological roles.

*An. stephensi* has recently invaded Ethiopia and poses a significant threat to ongoing malaria control efforts. Although this invasive species has been reported from the southern [55, 56], eastern [57, 58], and western [58] parts of the country, it was not detected in our survey of the mid-Rift Valley. This absence may be owing to gaps in survey coverage, and additional targeted entomological surveillance is likely needed to verify its presence or absence in this area.

The blood meal analysis, though limited by a small sample size (*n* = 71), and the technical constraints of Sanger sequencing in detecting mixed meals, provides crucial preliminary data on host preferences. The pronounced anthropophilic behavior observed in the *Cx. Pipiens* complex is a concerning finding that suggests a high potential for zoonotic pathogen transmission to humans, particularly WNV, as members of this group are competent vectors for this virus [48]. Depending on the biotype, *Cx. pipiens* can display ornithophilic, mammophilic, or anthropophilic behaviors [59]. However, the methodological limitations mean we likely underestimated the true zoophilic tendency and diversity of hosts. In contrast, *Cx. tritaeniorhynchus* demonstrated a broader range of host preferences, feeding on humans, equines, rodents, and cattle. Previous studies have documented this broad host range for *Cx. tritaeniorhynchus* [60, 61]. Meanwhile, *Ma. uniformis* is primarily zoophilic, aligning with our findings [62]. The diverse host ranges of *Cx. tritaeniorhynchus* and *Ma. uniformis* suggest their capacity to sustain pathogen transmission cycles among various vertebrate hosts. Future studies employing next-generation sequencing (NGS) on a larger sample size are essential to overcome these limitations and fully quantify the degree of mixed feeding and zoonotic connections in this ecosystem.

## Conclusions

This study offers valuable preliminary insights into the diversity of mosquito species, genetic variation, and host-feeding preferences within the Ethiopian Rift Valley. The findings emphasize the potential of molecular techniques to enhance traditional entomological methods and improve the accuracy of mosquito identification. While the study is limited in both geographic and temporal scope, it highlights mosquito species of medical and veterinary significance and suggests implications for arboviral disease surveillance. To develop a more comprehensive understanding of mosquito diversity in Ethiopia, future research should aim for broader spatial coverage, incorporate multiple sampling seasons, and utilize additional molecular markers.

## Data Availability

Comprehensive specimen data is available on BOLD: www.boldsystems.org under the dataset name DS-ETH1, titled “Barcoding Ethiopian Mosquitoes (Diptera, Culicidae).” The nucleotide sequences generated in this study have been deposited in the GenBank database under the accession nos. (PQ394790, PQ410334-345, PQ416592-601, PQ423764-768, PQ424700-705, PQ424992-5005, PQ425856-860, PQ428098-8126, PQ431193-196, PQ431958-962, PQ432462-483, PQ432493-514, PQ435392-398, PQ436010-020, PQ436021-025, PQ443031-034). The dataset can also be accessed using the link 10.5883/DS-ETH1.

## References

[1] Miller BR. Arboviruses. In: Mahy BWJ, Van Regenmortel MHV, editors. Encyclopedia of virology. Amsterdam: Elsevier; 2008.

[2] Madewell ZJ. Arboviruses and their vectors. South Med J. 2020;113:520–3 33005970 10.14423/SMJ.0000000000001152 PMC8055094

[3] Hubálek Z, Rudolf I, Nowotny N. Arboviruses pathogenic for domestic and wild animals. Adv Virus Res. 2014;89:201–75 24751197 10.1016/B978-0-12-800172-1.00005-7

[4] Wilder-Smith A, Gubler DJ, Weaver SC, Monath TP, Heymann DL, Scott TW. Epidemic arboviral diseases: priorities for research and public health. Lancet Infect Dis. 2017;17:101–6. 10.1016/S1473-3099(16)30518-7 28011234

[5] Leta S, Beyene TJ, De Clercq EM, Amenu K, Kraemer MUG, Revie CW. Global risk mapping for major diseases transmitted by *Aedes aegypti* and *Aedes albopictus*. Int J Infect Dis. 2018;67:25–35. 29196275 10.1016/j.ijid.2017.11.026 PMC5976855

[6] Kraemer MUG, Reiner RC, Brady OJ, Messina JP, Gilbert M, Pigott DM, et al. Past and future spread of the arbovirus vectors *Aedes aegypti* and *Aedes albopictus*. Nat Microbiol. 2019;4:854–63. 30833735 10.1038/s41564-019-0376-y PMC6522366

[7] Harbach RE. Mosquito taxonomic inventory 2024. https://mosquito-taxonomic-inventory.myspecies.info/. Accessed 23 Mar 2024.

[8] Bangoura ST, Sidibé S, Kaba L, Mbaye A, Hounmenou CG, Diallo A, et al. Seroprevalence of seven arboviruses of public health importance in sub-Saharan Africa: a systematic review and meta-analysis. BMJ Glob Heal. 2024;9:1–12. 10.1136/bmjgh-2024-016589 PMC11529691 39486798

[9] Hernández-Triana LM, Garza-Hernández JA, Ortega Morales AI, Prosser SWJ, Hebert PDN, Nikolova NI, et al. An integrated molecular approach to untangling host–vector–pathogen interactions in mosquitoes (Diptera: Culicidae) from sylvan communities in Mexico. Front Vet Sci. 2021;7:564791. 33778029 10.3389/fvets.2020.564791 PMC7988227

[10] Hill CA, Kafatos FC, Stansfield SK, Collins FH. Arthropod-borne diseases: vector control in the genomics era. Nat Rev Microbiol. 2005;3:262–8. 15703759 10.1038/nrmicro1101

[11] Krzywinski J, Besansky NJ. Molecular systematics of *Anopheles*: from subgenera to subpopulations. Annu Rev Entomol. 2003;48:111–39. 12208816 10.1146/annurev.ento.48.091801.112647

[12] Walton C, Sharpe RG, Pritchard SJ, Thelwell NJ, Butlin RK. Molecular identifi cation of mosquitospecies. Biol J Linn Soc. 1999;68:241–56.

[13] Noureldin E, Tan D, Daff alla O, Almutairi H, Ghzwani J, Torno M, et al. DNA Barcoding of PotentialMosquito Disease Vectors (Diptera, Culicidae) in Jazan Region, Saudi Arabia. Pathogens. 2022;11:1–15. 35631007 10.3390/pathogens11050486 PMC9171578

[14] Getachew D, Gebre-Michael T, Balkew M, Tekie H. Species composition, blood meal hosts and *Plasmodium* infection rates of *Anopheles* mosquitoes in Ghibe River Basin, southwestern Ethiopia. Parasit Vectors. 2019;12:1–15. 10.1186/s13071-019-3499-3. 31122286 10.1186/s13071-019-3499-3 PMC6533711

[15] Guta W, Simma EA, Yewhalaw D. Species composition, blood meal sources and insecticide susceptibility status of *Culex* mosquitoes from Jimma area, Ethiopia. Int J Trop Insect Sci. 2021;41:533–9.

[16] Jaleta MB, Tefera M, Negussie H, Mulatu T, Berhe T, Belete F, et al. Entomological survey of the potential vectors of rift valley fever virus and absence of detection of the virus genome from the vectors in various niches in the southern half of the Great Rift Valley of Ethiopia. Vet Med Sci. 2022;8:2716–25. 36104829 10.1002/vms3.941 PMC9677412

[17] Ashfaq M, Hebert PDN, Mirza JH, Khan AM, Zafar Y, Mirza MS. Analyzing mosquito (Diptera: Culicidae) diversity in Pakistan by DNA barcoding. PLoS ONE. 2014;9:1–12. 10.1371/journal.pone.0097268 PMC4036727 24827460

[18] Beebe NW. DNA barcoding mosquitoes: advice for potential prospectors. Parasitol. 2018;145:622–33. 10.1017/S0031182018000343 29564995

[19] Weeraratne TC, Surendran SN, Parakrama Karunaratne SHP. DNA barcoding of morphologically characterized mosquitoes belonging to the subfamily Culicinae from Sri Lanka. Parasit Vectors. 2018;11:266. 29695263 10.1186/s13071-018-2810-z PMC5918568

[20] Esayas E, Assefa M, Bennett A, Thomsen E, Gowelo S, Vajda E, et al. Bionomic characterization of *Anopheles* mosquitoes in the Ethiopian highlands and lowlands. Parasit Vectors. 2024;17:1–15. 10.1186/s13071-024-06378-3. 39014474 10.1186/s13071-024-06378-3 PMC11251230

[21] Carter TE, Yared S, Getachew D, Spear J, Choi SH, Samake JN, et al. Genetic diversity of *Anopheles stephensi* in Ethiopia provides insight into patterns of spread. Parasit Vectors. 2021;14:1–12. 10.1186/s13071-021-05097-3. 34895319 10.1186/s13071-021-05097-3 PMC8665610

[22] González MA, Bravo-Barriga D, Rodríguez-Sosa MA, Rueda J, Frontera E, Alarcón-Elbal PM. Species diversity, habitat distribution, and blood meal analysis of haematophagous dipterans collected by CDC-UV light traps in the Dominican Republic. Pathogens. 2022;11:714. 35889959 10.3390/pathogens11070714 PMC9319014

[23] Kent RJ. Molecular methods for arthropod bloodmeal identification and applications to ecological and vector-borne disease studies. Mol Ecol Resour. 2009;9:4–18. 21564560 10.1111/j.1755-0998.2008.02469.x

[24] Kitano T, Umetsu K, Tian W, Osawa M. Two universal primer sets for species identification among vertebrates. Int J Legal Med. 2007;121:423–7. 16845543 10.1007/s00414-006-0113-y

[25] Rozendaal JA. Vector control: methods for use by individuals and communities. Geneva: World Health Organization; 1997.

[26] Potter MA. WRBU: keys to the medically important mosquito species: identification key to the genera of adult mosquitoes for the world. Adapted from Harbach and Sandlant with the addition of Onirion and Verrallina. 2016.

[27] Edwards F. Mosquitoes of the Ethiopian Region. III. Culicine adults and pupae. London: CABI; 1941.

[28] Folmer O, Black M, Hoeh W, Lutz R, Vrijenhoek R. DNA primers for amplification of mitochondrial cytochrome c oxidase subunit I from diverse metazoan invertebrates. Mol Mar Biol Biotechnol. 1994;3:294–9. 7881515

[29] Ratnasingham S, Hebert PDN. Bold: the barcode of life data system. Mol Ecol Notes. 2007;7:355–64. 18784790 10.1111/j.1471-8286.2007.01678.x PMC1890991

[30] Benson DA, Cavanaugh M, Clark K, Karsch-Mizrachi I, Lipman DJ, Ostell J, et al. GenBank. NucleicAcids Res. 2013;41:D36–D42. 23193287 10.1093/nar/gks1195 PMC3531190

[31] Baena-Bejarano N, Reina C, Martínez-Revelo DE, Medina CA, Tovar E, Uribe-Soto S, et al. Taxonomic identification accuracy from BOLD and GenBank databases using over a thousand insect DNA barcodes from Colombia. PLoS ONE. 2023;18:1–19. 10.1371/journal.pone.0277379 PMC10124890 37093820

[32] Librado P, Rozas J. DnaSP v5: a software for comprehensive analysis of DNA polymorphism data. Bioinformatics. 2009;25:1451–2. 19346325 10.1093/bioinformatics/btp187

[33] O’Connor C. The distribution of Anopheline mosquitoes in Ethiopia. Mosq News. 1967;27:42–53.

[34] Adugna F, Wale M, Nibret E. Review of Anopheles Mosquito Species, Abundance, and Distribution inEthiopia. J Trop Med. 2021;2021:1–7. 34603455 10.1155/2021/6726622 PMC8486561

[35] Massebo F, Balkew M, Gebre-Michael T, Lindtjørn B. Zoophagic behaviour of Anopheline mosquitoes in southwest Ethiopia: opportunity for malaria vector control. Parasit Vectors. 2015;8:1–9. 10.1186/s13071-015-1264-9. 26684464 10.1186/s13071-015-1264-9 PMC4684615

[36] Li CX, Shi M, Tian JH, Lin XD, Kang YJ, Chen LJ, et al. Unprecedented genomic diversity of RNA viruses in arthropods reveals the ancestry of negative-sense RNA viruses. Elife. 2015;2015:1–26. 10.7554/eLife.05378 PMC4384744 25633976

[37] Zittra C, Flechl E, Kothmayer M, Vitecek S, Rossiter H, Zechmeister T, et al. Ecological characterization and molecular differentiation of *Culex pipiens* complex taxa and *Culex torrentium* in Eastern Austria. Parasit Vectors. 2016;9:1–9. 10.1186/s13071-016-1495-4. 27067139 10.1186/s13071-016-1495-4 PMC4828795

[38] Farajollahi A, Fonseca DM, Kramer LD, Marm KA. “Bird biting” mosquitoes and human disease: a review of the role of *Culex pipiens* complex mosquitoes in epidemiology. Infect Genet Evol. 2011;11:1577–85. 21875691 10.1016/j.meegid.2011.08.013 PMC3190018

[39] Fu YX. Statistical tests of neutrality of mutations against population growth, hitchhiking and background selection. Genetics. 1997;147:915–25. 9335623 10.1093/genetics/147.2.915 PMC1208208

[40] Smith JL, Fonseca DM. Rapid assays for identification of members of the *Culex* (*Culex*) *pipiens* complex, their hybrids, and other sibling species (Diptera: Culicidae). Am J Trop Med Hyg. 2004;70:339–45. 15100444

[41] Boddé M, Makunin A, Ayala D, Bouafou L, Diabaté A, Ekpo UF, et al. High-resolution species assignment of *Anopheles* mosquitoes using k-mer distances on targeted sequences. Elife. 2022;11:1–40. 10.7554/eLife.78775 PMC9648975 36222650

[42] Bursali F, Simsek FM. Population genetics of *Culex tritaeniorhynchus* (Diptera: Culicidae) in Türkiye. Acta Parasitol. 2024;69:1157–71. 38592372 10.1007/s11686-024-00844-9 PMC11182820

[43] Mwangangi JM, Mbogo CM, Orindi BO, Muturi EJ, Midega JT, Nzovu J, et al. Shifts in malaria vector species composition and transmission dynamics along the Kenyan coast over the past 20 years. Malar J. 2013;12:13. 23297732 10.1186/1475-2875-12-13 PMC3544599

[44] Goupeyou-Youmsi J, Rakotondranaivo T, Puchot N, Peterson I, Girod R, Vigan-Womas I, et al. Differential contribution of *Anopheles coustani* and *Anopheles arabiensis* to the transmission of *Plasmodium falciparum* and *Plasmodium vivax* in two neighbouring villages of Madagascar. Parasit Vectors. 2020;13:430. 32843082 10.1186/s13071-020-04282-0 PMC7447585

[45] Degefa T, Zeynudin A, Godesso A, Michael YH, Eba K, Zemene E, et al. Malaria incidence and assessment of entomological indices among resettled communities in Ethiopia: a longitudinal study. Malar J. 2015;14:24. 25626598 10.1186/s12936-014-0532-z PMC4318213

[46] Nepomichene TNJJ, Raharimalala FN, Andriamandimby SF, Ravalohery JP, Failloux AB, Heraud JM, et al. Vector competence of *Culex antennatus* and* Anopheles coustani* mosquitoes for Rift Valley fever virus in Madagascar. Med Vet Entomol. 2018;32:259–62. 29383746 10.1111/mve.12291

[47] Epelboin Y, Talaga S, Epelboin L, Dusfour I. Zika virus: an updated review of competent or naturally infected mosquitoes. PLoS Negl Trop Dis. 2017;11:e0005933. 10.1371/journal.pntd.0005933. 29145400 10.1371/journal.pntd.0005933 PMC5690600

[48] Fonseca DM, Keyghobadi N, Malcolm CA, Mehmet C, Schaffner F, Mogi M, et al. Emerging vectors in the *Culex pipiens* complex. Science. 2004;303:1535–8. 15001783 10.1126/science.1094247

[49] Tolsá-García MJ, Wehmeyer ML, Lühken R, Roiz D. Worldwide transmission and infection risk of mosquito vectors of West Nile, St. Louis encephalitis, Usutu and Japanese encephalitis viruses: a systematic review. Sci Rep. 2023;13:1–13. 10.1038/s41598-022-27236-1. 36609450 10.1038/s41598-022-27236-1 PMC9822987

[50] Mulvey P, Duong V, Boyer S, Burgess G, Williams DT, Dussart P, et al. The ecology and evolution of Japanese encephalitis virus. Pathogens. 2021;10:1–17. 10.3390/pathogens10121534 PMC8704921 34959489

[51] Azari-Hamidian S, Abai MR, Norouzi B. *Mansonia uniformis* (Diptera: Culicidae), a genus and species new to southwestern Asia, with a review of its medical and veterinary importance. Zootaxa. 2020;4772:385–95. 10.11646/zootaxa.4772.2.10 33055620

[52] Campbell LP, Reuman DC, Lutomiah J, Townsend Peterson A, Linthicum KJ, Britch SC, et al. Predicting abundances of *Aedes mcintoshi*, a primary Rift Valley fever virus mosquito vector. PLoS ONE. 2019;14:1–19. 10.1371/journal.pone.0226617. 10.1371/journal.pone.0226617 PMC6917266 31846495

[53] McPhatter L, Gerry AC. Effect of CO2 concentration on mosquito collection rate using odor-baited suction traps. J Vector Ecol. 2017;42:44–50. 28504452 10.1111/jvec.12238

[54] Valentine MJ, Ciraola B, Jacobs GR, Arnot C, Kelly PJ, Murdock CC. Effects of seasonality and land use on the diversity, relative abundance, and distribution of mosquitoes on St. Kitts, West Indies. Parasit Vectors. 2020;13:1–14. 10.1186/s13071-020-04421-7. 33138849 10.1186/s13071-020-04421-7 PMC7607626

[55] Massebo F, Ashine T, Negash N, Eligo N, Hailemeskel E, Minda TT, et al. The expansion of an invasivemalaria vector: Anopheles stephensi emergence in Arba Minch town in the southern Rift Valley ofEthiopia. Parasitol Res. 2024;123:333. 39331165 10.1007/s00436-024-08356-1 PMC11436467

[56] Hawaria D, Kibret S, Zhong D, Lee MC, Lelisa K, Bekele B, et al. First report of *Anopheles stephensi* from southern Ethiopia. Malar J. 2023;22:1–8. 10.1186/s12936-023-04813-x. 38066610 10.1186/s12936-023-04813-x PMC10704791

[57] Waymire E, Duddu S, Yared S, Getachew D, Dengela D, Bordenstein SR, et al. Wolbachia 16S rRNA haplotypes detected in wild *Anopheles stephensi *in eastern Ethiopia. Parasit Vectors. 2022;15:178. 35610655 10.1186/s13071-022-05293-9 PMC9128127

[58] Ashine T, Eyasu A, Asmamaw Y, Simma E, Zemene E, Epstein A, et al. Spatiotemporal distribution and bionomics of *Anopheles stephensi* in different eco-epidemiological settings in Ethiopia. Parasit Vectors. 2024;17:166. 38556881 10.1186/s13071-024-06243-3 PMC10983662

[59] Blom R, Krol L, Langezaal M, Schrama M, Trimbos KB, Wassenaar D, et al. Blood-feeding patterns of *Culex pipiens* biotype *pipiens* and *pipiens/molestus* hybrids in relation to avian community composition in urban habitats. Parasit Vectors. 2024;17:1–12. 10.1186/s13071-024-06186-9. 38424573 10.1186/s13071-024-06186-9 PMC10902945

[60] Fall AG, Diaïté A, Lancelot R, Tran A, Soti V, Etter E, et al. Feeding behaviour of potential vectors of West Nile virus in Senegal. Parasit Vectors. 2011;4:1–7. 21651763 10.1186/1756-3305-4-99 PMC3118230

[61] Arunachalam N, Samuel PP, Hiriyan J, Rajendran R, Dash AP. Short report: observations on the multiple feeding behavior of *Culex tritaeniorhynchus* (Diptera: Culicidae), the vector of Japanese encephalitis in Kerala in southern India. Am J Trop Med Hyg. 2005;72:198–200. 15741557

[62] Wilson JJ, Sevarkodiyone SP. Host preference of blood feeding mosquitoes in rural areas of southern Tamil Nadu India. Acad J Entomol. 2015;8:80–3.

